# Eye-Movement–Based Assessment of the Perceptual Consequences of Glaucomatous and Neuro-Ophthalmological Visual Field Defects

**DOI:** 10.1167/tvst.10.2.1

**Published:** 2021-02-05

**Authors:** Rijul Saurabh Soans, Alessandro Grillini, Rohit Saxena, Remco J. Renken, Tapan Kumar Gandhi, Frans W. Cornelissen

**Affiliations:** 1 Department of Electrical Engineering, Indian Institute of Technology – Delhi, New Delhi, India; 2 Laboratory of Experimental Ophthalmology, University Medical Center Groningen, University of Groningen, The Netherlands; 3 Department of Ophthalmology, Dr. Rajendra Prasad Centre for Ophthalmic Sciences, All India Institute of Medical Sciences, New Delhi, India; 4 Cognitive Neuroscience Center, Department of Biomedical Sciences of Cells and Systems, University Medical Center Groningen, University of Groningen, The Netherlands

**Keywords:** visual field defects, glaucoma, neuro-ophthalmology, perimetry, eye movements, eye tracking, screening, decision trees, cross correlogram

## Abstract

**Purpose:**

Assessing the presence of visual field defects (VFD) through procedures such as perimetry is an essential aspect of the management and diagnosis of ocular disorders. However, even the latest perimetric methods have shortcomings—a high cognitive demand and requiring prolonged stable fixation and feedback through a button response. Consequently, an approach using eye movements (EM)—as a natural response—has been proposed as an alternate way to evaluate the presence of VFD. This approach has given good results for computer-simulated VFD. However, its use in patients is not well documented yet. Here we use this new approach to quantify the spatiotemporal properties (STP) of EM of various patients suffering from glaucoma and neuro-ophthalmological VFD and controls.

**Methods:**

In total, 15 glaucoma patients, 37 patients with a neuro-ophthalmological disorder, and 21 controls performed a visual tracking task while their EM were being recorded. Subsequently, the STP of EM were quantified using a cross-correlogram analysis. Decision trees were used to identify the relevant STP and classify the populations.

**Results:**

We achieved a classification accuracy of 94.5% (TPR/sensitivity = 96%, TNR/specificity = 90%) between patients and controls. Individually, the algorithm achieved an accuracy of 86.3% (TPR for neuro-ophthalmology [97%], glaucoma [60%], and controls [86%]). The STP of EM were highly similar across two different control cohorts.

**Conclusions:**

In an ocular tracking task, patients with VFD due to different underlying pathology make EM with distinctive STP. These properties are interpretable based on different clinical characteristics of patients and can be used for patient classification.

**Translational Relevance:**

Our EM-based screening tool may complement existing perimetric techniques in clinical practice.

## Introduction

Assessing the presence of visual field defects (VFD) is a critical aspect of diagnosing the presence of many ocular disorders such as glaucoma and age-related macular degeneration. In the context of neuro-ophthalmology, VFD are useful to localize the site of lesions in the visual pathway. Besides this, the visual fields are helpful to monitor patients with recurring neuro-ocular diseases such as pituitary adenomas or optic neuropathy and to understand their visual abilities on daily-life tasks during rehabilitation.^1^

The most common methods to determine the presence of VFD are standard automated perimetry (SAP) and frequency doubling technology (FDT) perimetry. Although clinically very useful, these standard methods have some limitations that can prevent a proper visual field assessment in various clinical populations, such as the elderly, people with cognitive impairments, and young children. We will address these limitations below in more detail.

If a new method could overcome these limitations, it could be used to screen for VFD also in such populations. In this study, we ask whether a recent approach based on analyzing eye-movements (EM) made during a tracking task could fulfill this role.^2^^–^^4^ Specifically, we will quantify the spatiotemporal properties (STP) of EM of a group of patients suffering from glaucoma or neuro-ophthalmologic disorders. Subsequently, we will determine whether classifying these properties can be used to reliably screen for VFD.

SAP has a number of limitations that render it problematic for various patient groups. It is cognitively demanding—for example, the patient has to keep their eyes fixated on a central cross for nearly 10 minutes while a small light is being projected at them. Although recent protocols such as SITA-Faster (Swedish Interactive Thresholding Algorithm) can shorten this duration, it can still be discomforting because one's natural reflex is to look toward any suddenly-appearing visual stimulus. Moreover, the technique requires the patient to press a button upon perceiving the stimulus. The cognitive and task demands imply that there is a learning curve associated with performing the test, which may interfere with the goal of catching the disorder. Although the associated false-positive and false-negative responses are informative about performance in a way, they increase test duration and may reduce the reliability of the results. FDT perimetry, on the other hand, requires much less time. However, as with SAP, the method demands stable fixation and manual responding. Furthermore, FDT perimetry is poor at differentiating hemianopic and glaucomatous VFD.^5^

Methods such as SAP and FDT rely on discrete and trial-based psychophysical methods wherein the subject presses a button if they perceive the stimulus. However, in daily life, the response to a natural stimulus is seldom discrete. Rather, it is in accordance with the statistical structure and dynamics of the stimulus.^6^ Prior studies^3^^,^^7^ therefore have turned to continuous tracking of stimulus position to model behavior. For example, it is possible to derive the sensitivity of detecting a stimulus from someone's ability to track that stimulus using a computer mouse or joystick.^3^^,^^8^^,^^9^ The need for such a conscious manual response can be eliminated by tracking EM.^10^ EM are intuitive to make—if a person detects a suddenly-appearing visual stimulus in a particular position, they are inclined to direct their eyes to that location. Consequently, a continuous assessment of EM in a tracking paradigm has the potential to overcome some of the limitations of SAP/FDT.

For this reason, we have previously developed an approach in which EM were measured while participants performed a continuous stimulus-tracking task.^3^ The Eye-Movement Correlogram (EMC) method^2^ was subsequently applied to the data for quantifying the STP of the EM. The intuition behind this spatiotemporal approach is the following: if a participant's visual sensitivity is reduced due to the presence of a VFD, then one can expect temporal delays in their smooth pursuit of a target. Moreover, if the participant is unable to track a stimulus because of a VFD blocking their view on it, they must search for it—which will result in even longer temporal delays and increased spatial errors. Furthermore, VFDs can be expected to affect the process of EM generation because of alterations in the balance between saccade generation and fixation stabilization.^11^

To verify these aspects, the approach was applied to classify participants with computer-simulated VFD mimicking the acute effects of glaucoma, age-related macular disease and hemianopia.^4^ It provided good classification results (accuracy = 90%; true positive rate [TPR] = 98%). However, while this provides proof that the method could work in principle, it does not guarantee that it will be useful in patients as there could be a difference in the EM behavior of participants with simulated and real scotomas,^11^ for example, because of long-term adaptation or the learning of compensatory strategies.

In this article, we hypothesize that the STP of EM in a visual-tracking task will be systematically and categorically different for three groups of participants: patients with glaucoma, patients with a neuro-ophthalmologic disorder, and healthy controls.

The procedure used in this article to test this hypothesis can be summarized as follows: First, the participants track a randomly moving blob on the screen while their EM are being monitored through an eye tracker. Next, we extract the STP of their EM using the EMC technique. Then, to verify the stability of our approach across ethnicity, we compare the STP of our participants to those of a cohort of Caucasian participants. Subsequently, we train a decision tree algorithm to classify the VFD based on the obtained features in our participations. Finally, we try to understand the observed STP in each of the patient groups as a consequence of their pathology.

## Methods

### Ethical Approval

The ethics board of the All India Institute of Medical Sciences–Delhi (AIIMS) and Indian Institute of Technology–Delhi (IITD) approved this study. All participants provided written informed consent before participation. The study adhered to the tenets of the Declaration of Helsinki.

### Participants

Nineteen patients with glaucoma, 43 patients with a neuro-ophthalmologic disorder, and 21 controls volunteered to participate. All patients with glaucoma and neuro-ophthalmologic disorder were recruited from AIIMS. The inclusion criteria for the patient group were diagnosed patients with neuro-ophthalmologic disorders or glaucoma with best-corrected visual acuity (BCVA) of 6/36 or better and having stable and reliable visual fields. The inclusion criteria for the controls were having intact visual fields with BCVA of 6/9 (0.67 or ≤0.17 logMAR) or better in both eyes. The exclusion criteria for the patients were having amblyopia, nystagmus, strabismus, or any conditions that affect the extraocular muscles. The exclusion criteria for both groups were subjects below the age of 18 years, subjects with astigmatism higher than two diopters. The glaucoma patient group included participants with primary open angle glaucoma (POAG), steroid-induced glaucoma, juvenile open-angle glaucoma, and primary angle closure glaucoma. The neuro-ophthalmologic disorder group included participants with temporal disc pallor, toxic optic neuropathy, optic neuritis, pituitary adenoma, temporal optic nerve pallor, secondary optic atrophy, papilledema, idiopathic intracranial hypertension, and nonarteritic anterior ischemic optic neuropathy. Table 1 shows the demographics of all the included participants. Six participants with neuro-ophthalmologic VFD and four participants with glaucoma were excluded because of poor eye tracking calibration and data. In total, 15 patients with glaucoma, 37 patients with neuro-ophthalmologic VFD, and 21 controls were included in this study. Additionally, to verify the stability of the method across ethnicity, we also included data from 48 Dutch controls performing the same experiment (eight observers per decade; age-range: 30–79 years; four male per decade). The data were collected at Royal Dutch Visio, Haren, The Netherlands.^12^

**Table 1.** Group Demographics and Clinical Characteristics

Characteristics	Neuro-Ophthalmologic Disorders (*N* = 37)	Glaucoma (*N* = 15)	Controls (*N* = 21)
Age (y)	36.9 (12.5)	43.9 (12.5)	38 (15.05)
Age Range (y)	18–73	24–61	20–67
Male (sex)	15 (40.5)	10 (66.6)	15 (71.4)
BCVA	0.78 (0.27)	0.72 (0.27)	0.89 (0.1)
BCVA Range	0.25–1	0.25–1	0.7–1
Subtypes	Temporal disc pallor (*n* = 7)	POAG (*n* = 9)	
	Toxic optic neuropathy (*n* = 5)	Steroid-induced glaucoma (*n* = 2)	
	Optic neuritis (*n* = 4)	JOAG (*n* = 3)	
	Pituitary adenoma (*n* = 6)	PACG (*n* = 1)	
	TONP (*n* = 3)		
	Secondary optic atrophy (*n* = 2)		
	Papilloedema (*n* = 4)		
	IIH (*n* = 2)		
	NAION (*n* = 2)		
	Brain infarction with parietal lobe involvement (*n* = 1)		
	Brain infarction with occipital lobe involvement (*n* = 1)		

Values are represented as mean (SD) or number (%). The *P* value for the differences in age group means for the three groups was 0.22.

IIH, idiopathic intracranial hypertension; JOAG, juvenile open-angle glaucoma; NAION, nonarteritic anterior ischemic optic neuropathy; PACG, primary angle closure glaucoma; TONP, temporal optic nerve pallor.

### Ophthalmic Data

All participants were refracted, and the BCVA was documented using a Snellen chart with optimal correction for the viewing distance. The BCVA (in decimal units) of the three groups is shown in Table 1. This was followed by a visual field assessment for each eye on a Humphrey Field Analyzer (HFA; Carl Zeiss Meditec, Jena, Germany) using the 30-2 grid, Goldman III stimulus and the Swedish Interactive Threshold Algorithm Fast (SITA-Fast). Tables 2 and 3,^4^ show the different types of VFD observed in the neuro-ophthalmology and glaucoma patient groups. Based on the Hodapp-Parrish-Anderson classification^13^ of the worse eye, the glaucoma group comprised five early-stage, three moderate, two advanced and five severe patients, and the neuro-ophthalmology group had eleven early-stage, nine moderate, thirteen advanced and four severe patients.

**Table 2.** Visual Field Defects Observed in the Neuro-Ophthalmology Category

Visual Field Defects	No. of Eyes (*n* = 64)
Hemianopia	10
Generalized constriction	10
Paracentral scotoma	9
Within normal limits	8
Enlargement of blind spot	8
Peripheral scotomas	6
Quadrantanopia	4
Altitudinal	4
Bitemporal hemianopia	2
Biarcuate	2
Central scotoma	1

**Table 3.** Visual Field Defects Observed in the Glaucoma Category

Visual Field Defects	No. of Eyes (*n* = 28)
Paracentral scotoma	7
Generalized constriction with central or temporal sparing	6
Arcuate	6
Within normal limits	4
Generalized constriction	3
Biarcuate	2

**Table 4.** A Description of the Spatiotemporal Properties Along With Their Corresponding Ranges

Category	Property Name	Description	Range
Spatial	1. PED: Amplitude	Describes the most frequent positional error observed. Higher values of amplitude for a mean of zero indicates better performance.	[0 1]
	2. PED: Mean	Describes the spatial offset. Values (in visual degrees) closer to zero indicate better performance.	[0 ∞]
	3. PED: Standard Deviation	Describes the spatial uncertainty: the spread of the positional deviations. Lower values indicate better performance.	[0 ∞]
	4. PED: adjusted *R*^2^	Describes how close the positional error distribution resembles a Gaussian distribution. Values closer to 1 indicate better performance.	≤1
Temporal	5. Average Velocity CCG: Amplitude	Shows the maximum correlation between the stimuli and gaze velocities. Higher values indicate better performance.	[−1 1]
	6. Average Velocity CCG: Mean	Describes the temporal lag between stimuli and gaze velocities (in ms). Lower values indicate better performance.	[0 ∞]
	7. Average Velocity CCG: Standard Deviation	Describes the temporal uncertainty: the time window (in ms) in which the observer is uncertain in their ability to track the stimulus. Lower values indicate better performance.	[0 ∞]
	8. Average Velocity CCG: Adjusted R-squared	Describes how close the temporal tracking performance resembles a Gaussian distribution. Values closer to 1 indicate better performance.	≤1
Integrated	9. Observation noise variance	Describes the noise internal to the observer Sensory noise estimated by measuring the variance of the observational noise using a flipped Kalman filter. Lower values indicate better STP.	[0 ∞]
	10. Similarity	Cosine similarity between gaze and stimulus vectors of positions. Higher values indicate better STP.	[0 1]

CCG, cross-correlogram; PED, positional error distribution.

### Stimulus and Eye Tracking Apparatus

The experiment was designed and conducted with custom made scripts in MATLAB R2018b using the Psychtoolbox^14^^,^^15^ and the Tobii Pro Software Development Kit (Tobii, Stockholm, Sweden). The gaze positions were acquired with a screen-based Tobii T120 eye-tracker (Tobii, Stockholm, Sweden) with a sampling frequency of 120 Hz, down-sampled to 60 Hz to match the refresh rate of the stimulus display monitor of the Tobii T120 eye tracker. The task was done monocularly using a Tobii Infrared-transparent occluder so that the eye tracker could monitor the gaze position unhindered while occluding the subjects’ eye.

The stimulus was a Gaussian luminance blob of 0.43° in diameter (equivalent to Goldman size III) moving according to a Gaussian random walk on the screen. The luminance blob was presented at a peak luminance of ∼ 165 cd/m^2^ on a uniform grey background (∼ 150 cd/m^2^), effectively having a contrast of 5% from the background. The 2D random-walk path had two modes: (1) the blob could move in a “smooth” mode where it moves continuously or (2) “displaced” mode where the blob jumps randomly to a new location on the screen every two seconds. The participants were seated comfortably and asked to place their chin on a chin-rest placed at a distance of 60 cm from the screen (with integrated eye tracker). Next, a five-point custom-made eye calibration was performed. Subsequently, participants were asked to “follow the moving blob.” They were allowed to blink as required. The experiment consisted of six trials of 20 seconds each. A participant could take a short break at the end of a trial if they felt a need for this.

### Eye-Tracking Data Pre-Processing

We first obtain the eye positions from the eye tracker in terms of screen coordinates. These positions are then converted to visual field coordinates. Subsequently, we correct for eye blinks by the following:

1. We differentiate the eye positions to obtain the horizontal and vertical gaze velocities.
2. In the vertical gaze velocity, we mark all spikes which go higher than the threshold of 190°/sec, and that are followed either by a flat line (first derivative of vertical gaze velocity is 0) or some missing data. These portions of the time series occur due to how video-based eye trackers record eyeblinks, that is, they wrongly interpret it as the pupil suddenly shifting upward when the eyelids close.
3. Next, we record the last valid position and dilate the blink by five samples before and after the period to define the beginning and the end of the blink period.
4. Finally, we fill the missing data with estimates inferred from forward and reverse autoregressive fits^16^ of 10 samples preceding and after the defined blink period.

Note that we discard a trial if the data losses caused by blinks or missing data exceed more than 33% of that entire trial's duration.

### Eye Movement Correlogram

Once the blink-filtered signal is obtained, we proceed to perform what is known as the EMC. A detailed treatment on the topic is available in Mulligan et al.^2^ We briefly describe the procedure here. EMC is an analytical tool that can be used to quantify the spatial and temporal relationships between the time series of a target stimulus and the corresponding response. It involves correlating the two time-series as a function of the time-lag between them. In our experiment, we apply the EMC technique to the stimulus and eye response velocities to quantify the *temporal* features of the EM. Because our experimental paradigm measures the horizontal and vertical components of the eye positions, each trial yields a “Horizontal Cross-Correlogram” and a “Vertical Cross-Correlogram,” respectively. Below, we describe the STP of EM in detail.

### STP

We distinguish between three categories of spatiotemporal properties:

A. Spatial Properties: These refer to the spatial accuracy of a participant's EM behavior and are quantified as follows:

(1) For every trial, we obtain the positional deviations between the stimulus and eye positions at each time instant.
(2) Next, we concatenate the positional errors of all the trials and obtain a probability distribution of the spatial errors across all trials.
(3) Subsequently, we fit a 1D Gaussian model to this probability distribution. This is the positional error distribution.
(4) Finally, we obtain the spatial properties from the parameters of the Gaussian model, that is, amplitude, mean (*μ*), standard deviation (SD) (*σ*) and variance explained (adjusted *R^2^*).

B. Temporal Properties: These are the parameters that describe the temporal aspects of EM and are obtained as follows:

1) For every trial, we first differentiate the stimulus positions and the gaze positions with respect to time to obtain their corresponding velocities.
2) Next, we perform a normalized cross-correlation between the stimulus and the gaze velocities as a function of the time-lag. The time lag varies from −1 to +1 seconds with a step-size of 0.016 (i.e., the interframe interval).
3) Each trial produces two cross-correlograms (CCG) – one each for the horizontal and vertical components of the stimulus and eye response.
4) Then, we average all the CCGs obtained over the number of trials. Furthermore, we fit a one-dimensional Gaussian model to the averaged CCG.
5) Finally, we obtain the temporal properties from the parameters of the Gaussian model, that is, amplitude, mean (*μ*), standard deviation (*σ*), and variance explained (adjusted *R^2^*).

C. Integrated STP: These properties capture both the spatial errors and the temporal delays of the eye movements with respect to the stimulus. We consider two such properties:

1) *Cosine Similarity*: It describes the amount of similarity between the stimulus and the gaze positions, irrespective of their length. This metric is useful as sometimes the stimulus and gaze position vectors may be far in terms of their Euclidean distances, but could be much closer in terms of their orientations. It is computed as follows:
  (1) $$\begin{array}{c} \begin{array}{c} \mathrm{Cosine}\mathrm{Similarity}=\frac{Stimulus\;Positions^{T}Gaze\;Positions}{\left|Stimulus\;Positions∥Gaze\;Positions\right|}, \end{array}\;\;\;\; \end{array}$$
  where *T* stands for transpose of the vector containing the stimulus positions and |.| refers to the magnitude of the vector.
2) *Observation Noise Variance*: Traditionally, Kalman filters have two sources of variance present in them: (1) Target displacement variance, that is, the variance associated with driving the target position at each time step and (2) observation noise variance—the variance associated with the present sensory observation.^3^ Here we use the latter as an estimate of the participant's spatiotemporal tracking abilities. If the stimulus is perceived well by the participant, then the gaze (observation) noise variance is low as compared to the stimulus displacement variance. This would mean that the difference between the prior stimuli position estimate and the current noisy gaze data is mostly due to changes in the position of the stimuli. Consequently, the previous estimate is weighted lower than the current observation. Given that we already know the stimuli positions and the corresponding gaze observations, we “overturn” the Kalman filter and estimate the noise parameter instead. The STP along with their corresponding ranges are summarized in Table 4.

### Test for the Stability of the STPs Across Ethnicity

Once the STP were obtained from the data, we first wanted to check whether ethnicity has an effect on these features, that is, whether the STP of EM of Indian controls would differ from those of Dutch controls. To test this out, we computed the modified Z-scores of each of the STP in the Indian control group against the Dutch normative group. The reason behind using modified Z-scores instead of the standard Z-score is because the former uses the median as a measure of deviation instead of the mean. Modified Z-scores are therefore less influenced by outliers and more robust.^17^ They are computed as follows:

(2) $$M_{i}=\frac{x_{i}-\bar{x}}{1.486\;MAD},$$

where $\bar{x}$ is the median of the data and *MAD* refers to the Median Absolute Deviation, which is further defined as:

(3) $$\mathrm{MAD}=\mathrm{median}\left(\left|Y_{i}-\bar{Y}\right|\right),$$

where $\bar{Y}$ refers to the median of the data.

Note that the experimental stimulus at Royal Dutch Visio was similar to that shown in AIIMS. Participants viewed both modes of the experiment on a Tobii 60 XL (Tobii, Stockholm, Sweden) eye tracker at a distance of 60 cm. The stimulus was a Gaussian luminance blob of 0.42° moving in a two-dimensional Gaussian random walk. The blob had a luminance of ∼160 cd/m^2^ against a uniform gray background (∼140 cd/m^2^)—which effectively had a Weber contrast of 5% from the background.

### Feature Selection and Participant Classification

After extracting the STP from the EM data, we proceed to train a decision tree (DT)^18^ classification model to discriminate between the different categories of patients and controls. They are constructed by splitting a set of labelled features (feature-space) at every “node” into smaller branches (sub-spaces) before converging onto a “class” or decision—called the “leaf.” In our case, the DTs begin with a 40-dimension feature space, that is, from the 40 STPs (10 each × horizontal & vertical components × for “smooth” and “displaced” modes). Subsequently, they split into smaller branches based on the “Gini's diversity index” (GDI) impurity criterion—a measure of homogeneity of the class labels reaching a particular node. The GDI is computed as follows:

(4) $$1-\sum f^{2}\left(i\right),$$

where *f(i)* is the fraction of the number of examples in the dataset for class *i* (with *i* being an indicator for control, glaucoma, and neuro-ophthalmologic disorder groups) arriving at a specific node. The GDI at a node returns a value of 0 indicating “purity” if it has samples of the same class, and the node becomes a leaf, thereby completing the decision. On the other hand, the GDI returns a positive value if there are samples from different classes arriving at a node. The node is then termed “impure,” and the node is subsequently split to minimize the GDI to converge onto a leaf eventually. The parameters of the model, such as the maximum number of tree splits and depth of the decision tree were tuned according to a 10-fold cross-validation technique. Here, the data are first partitioned to 10 folds. Subsequently, at every stage, one of the folds is used for testing while the remaining nine are used for training. The parameters leading to the least error across all the folds are then used to build the final model on our dataset. This technique is used to prevent overfitting and is useful for relatively small datasets such as ours, where we would like to maximize our error estimation.^19^

### Performance Metrics

The metrics evaluating the patient-categorization model are described below:

1) Sensitivity/true-positive rate (TPR): It refers to the proportion of subjects who test positive in a particular category among all the subjects who actually have the condition.
  (5) $$\begin{array}{c} \begin{array}{c} \mathrm{TP}R_{\mathrm{class}}=\frac{No.\;of\;true\;positives}{No.\;of\;true\;positives+No.\;of\;false\;negatives} \end{array}\;\;\;\; \end{array}$$
2) Specificity/true-negative rate (TNR): It refers to the proportion of subjects who test negative in a particular category among all the subjects who actually do not have the condition.
  (6) $$\begin{array}{c} \begin{array}{c} \mathrm{TN}R_{\mathrm{class}}=\frac{No.\;of\;true\;negatives}{No.\;of\;true\;negatives+No.\;of\;false\;positives} \end{array}\;\;\;\; \end{array}$$
3) Positive predictive value (PPV): It refers to the probability that a subject in a specific class truly has the specific condition, given that a positive result has already been seen.
  (7) $$\begin{array}{c} \begin{array}{c} \mathrm{PP}V_{\mathrm{class}}=\frac{No.\;of\;true\;positives}{No.\;of\;true\;positives+No.\;of\;false\;positives} \end{array}\;\; \end{array}$$
4) Negative predictive value (NPV): It refers to the probability that a subject in a specific class truly does not have the specific condition, given that a negative test result has already been seen.
  (8) $$\begin{array}{c} \begin{array}{c} \mathrm{NP}V_{\mathrm{class}}=\frac{No.\;of\;true\;negatives}{No.\;of\;true\;negatives+No.\;of\;false\;negatives} \end{array}\; \end{array}$$

## Results

To summarize the results, we found that the STP of EM are consistent across Indian and Dutch controls. Subsequently, we note that the neuro-ophthalmic patients had the highest average smooth pursuit latency as compared to glaucoma and the control groups. In terms of the positional error distributions, these patients also had higher standard deviations (positional and temporal uncertainties) for both the “smooth” and the “displaced” modes. The glaucoma group of patients, on the other hand, had a higher average latency compared to the rest of the groups in the “displaced” mode. Furthermore, we achieved a classification accuracy of 94.5% (TPR/sensitivity = 96%, TNR/specificity = 90%) between the patients and controls by constructing a screening model that uses STP of EM and decision trees. Finally, in terms of individual categorization into one of the three groups, the machine-learning algorithm achieved an accuracy of 86.3% (TPR for neuro-ophthalmology = 97%, glaucoma = 60%, and controls = 86%). We describe these results in more detail below.

### STP of Different Groups

**Figure 1.**
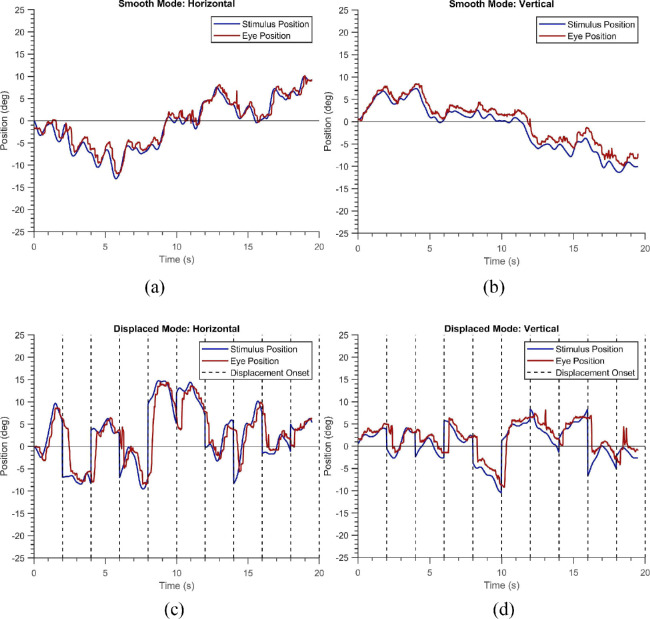
Examples of a single trial of a healthy participant in the “Smooth” (**a, b**) and the “Displaced” (**c, d**) modes. The movement of the stimuli is shown in *blue*, and the participant's gaze positions are shown in *red*. (**a, c**) Horizontal components of the stimuli and gaze positions. (**b, d**) The corresponding vertical components.

**Figure 2.**
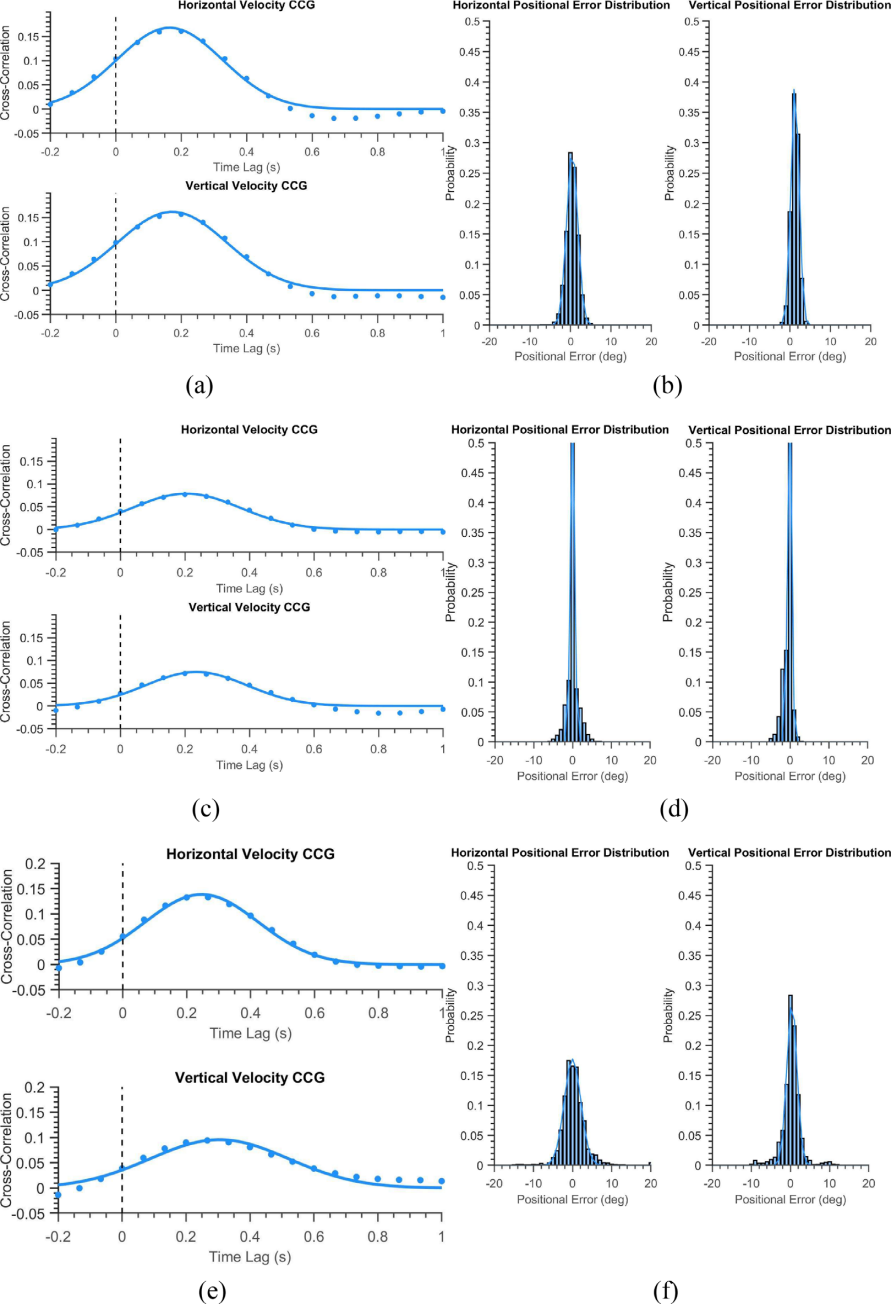
(**a, c, e**) The average velocity cross-correlograms for a healthy, a glaucoma and a neuro-ophthalmological participant in the “Smooth Mode” (*blue*), respectively. (**b, d, f**) The positional error distributions for the corresponding three individuals.

**Figure 3.**
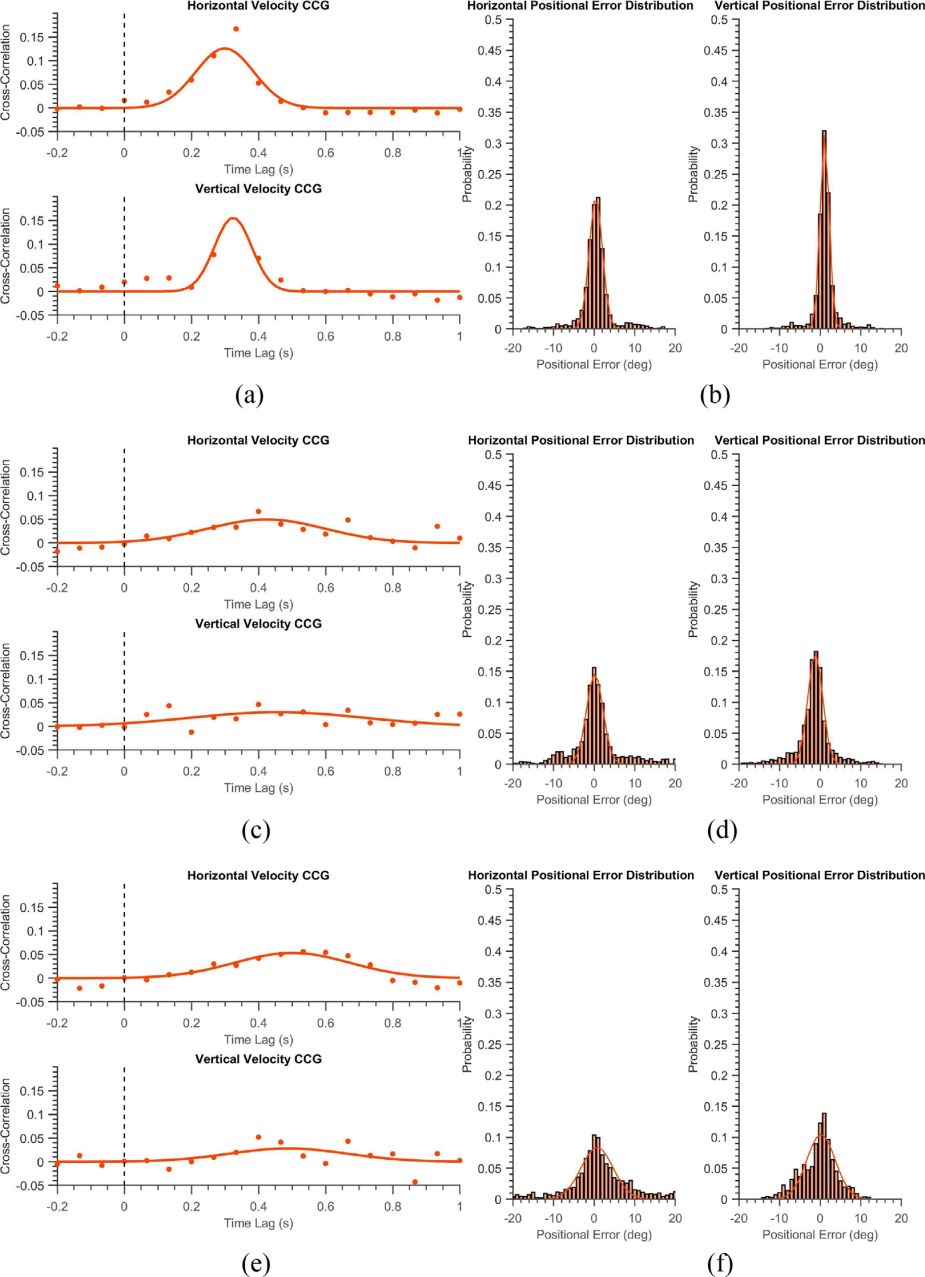
(**a, c, e**) The average velocity cross-correlograms for the same (the healthy, the glaucoma, and the neuro-ophthalmologic) participant shown in Figure 2 in the “Displaced Mode” (*orange*). (**b, d, f**) The positional error distributions for the corresponding three individuals.

Table 1 summarizes the demographics of the three groups. They did not differ significantly in age. Figure 1 shows six trials of a healthy participant in the “smooth” and the “displaced” mode. The position of the blob is depicted in blue while the eye positions are shown in red. The left panel of the figure shows the horizontal components of the stimuli and eye positions, while the right panel shows the corresponding vertical positions. One can notice visually that the blue and red time-series signals almost overlap with one another, indicating that the subject has followed the stimuli reasonably well. Figure 2a shows the normalized average cross-correlogram for the same subject. We observe a positive peak at a latency of 0.16 seconds (or 160 milliseconds) for the horizontal component and 0.17 seconds for the vertical one. Figure 2b shows the positional error distribution of the healthy subject - a narrow bell-shaped curve which has large values near zero-error - which is expected in a typical healthy subject. A curve fit to this distribution is used for subsequent analysis. Figures (3a & 3b) depict the STP as mentioned above of the healthy subject in the “displaced” mode. Given that the participant has to keep making saccades to keep up with the jumping blob, the latencies are a bit more (0.29 secs and 0.32 secs) as compared to those in the “smooth” mode. However, the positional error distributions still retain their shallow tails.

Figures 2c and 2d show that in the “smooth” mode, the glaucoma subject performs similar to the control subject with low temporal lags (0.2 secs and 0.23 secs) and positional uncertainty values (0.73 and 0.72) in the error distributions. However, as seen in Figures 3c and 3d, it is the “displaced mode” where the subject gets flagged into the glaucoma group of patients with the CCG showing substantial temporal delay (0.42 seconds and 0.45 seconds) and the positional error distribution having large standard deviation (positional uncertainty) (2.88 and 2.58) with fatter tails.

**Figure 4.**
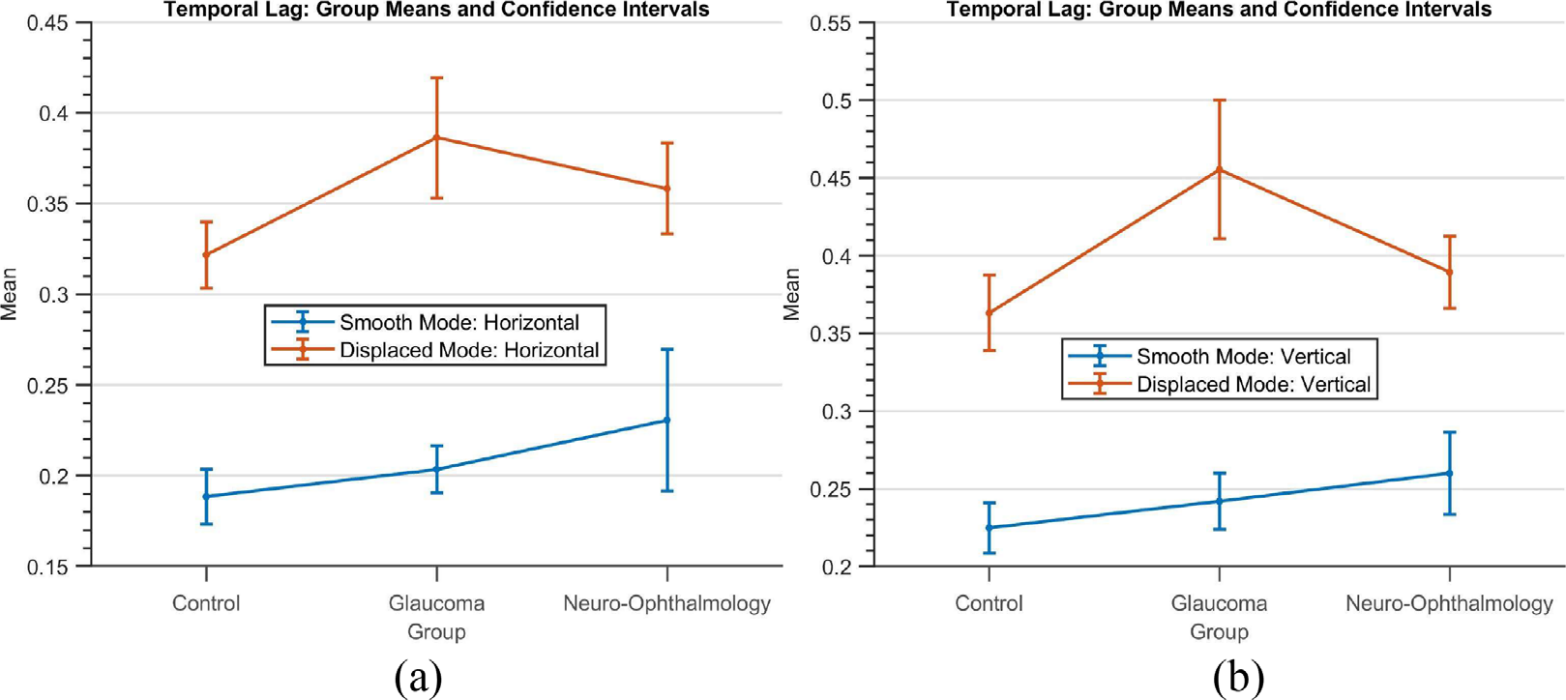
The group means and the corresponding 95% confidence intervals of the feature *Temporal Lag* for all the three groups.

Figure 4 shows the group means and the 95% confidence intervals of the three groups. Here, we see that when the blob moved smoothly, the mean temporal lag of glaucoma patients was higher (0.2 seconds and 0.24 seconds) than the controls (0.18 secs and 0.22 secs) but lower than the neuro-ophthalmic group of patients (0.23 seconds and 0.26 seconds) for the horizontal and vertical gaze components, respectively. However, when the blob jumped randomly every two seconds, the glaucoma group had the highest mean temporal lag across all subjects (i.e., 0.38 seconds and 0.45 seconds) as compared to the neuro-ophthalmic group (0.35 seconds and 0.38 seconds) and the controls (0.32 seconds and 0.36 seconds).

Figures 2e and 2f show a typical neuro-ophthalmic patient exhibiting more considerable delays during smooth pursuit (0.24 seconds and 0.3 seconds) than the rest of the showcased subjects. In the “displaced mode,” the patient exhibits a large spatial uncertainty (5.55 and 4.95) as depicted by the standard deviation of the flat positional error distributions.

### Test for Stability Across Ethnicity

**Figure 5.**
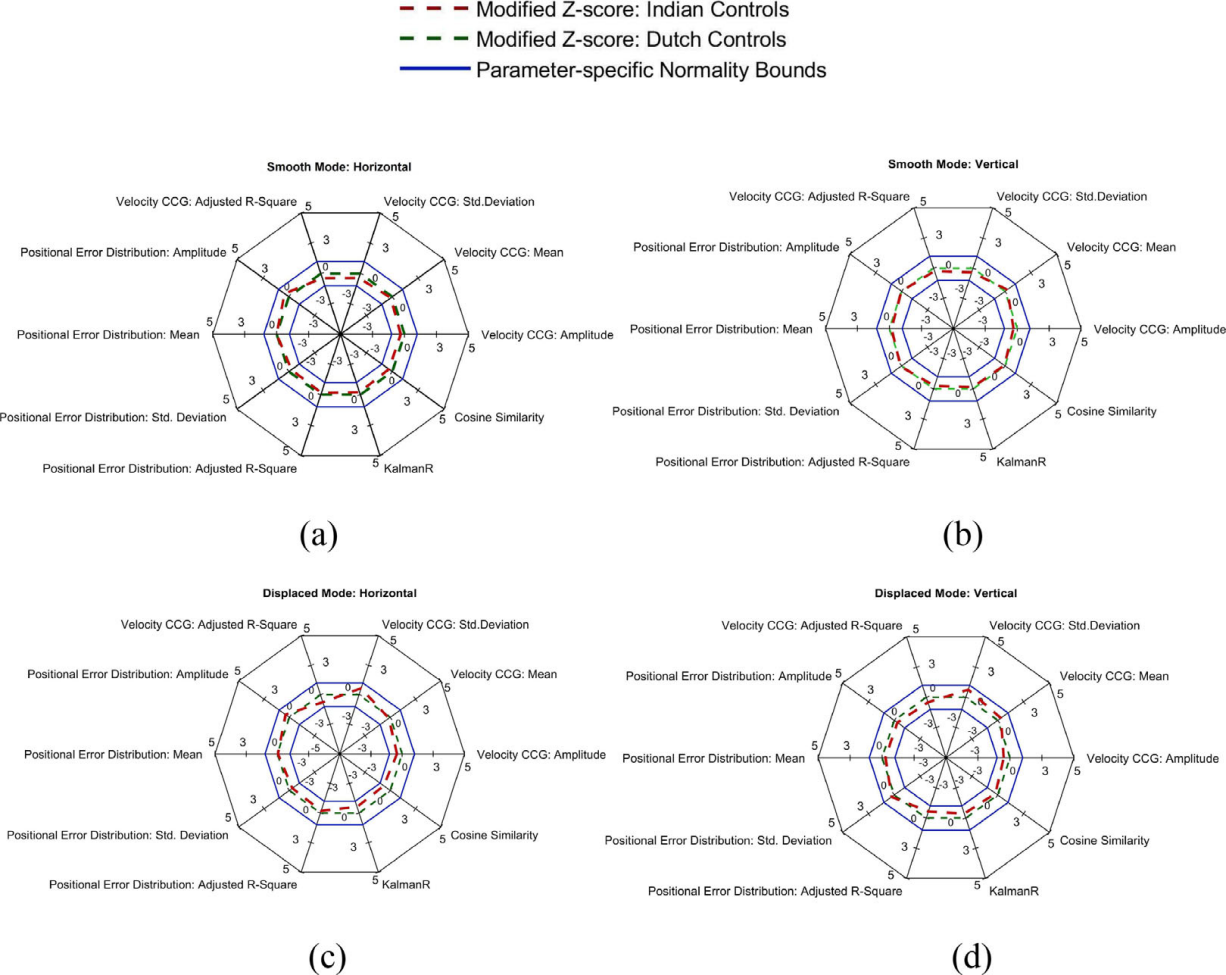
Spider plots depicting the modified Z-scores of the spatiotemporal properties of the horizontal and vertical eye components for Indian against the Dutch control cohort. (**a, b**, “Smooth Mode”; **c, d**, “Displaced Mode”).

Figure 5 shows the spider plots depicting the modified Z-scores of all the STP in both the experiment modes for the two ethnic control cohorts. A particular feature is considered to be an outlier if it differs from the normative population by ± 2 SD from the median. However, as seen in the figure, the spatiotemporal parameters of the EM made by Indian controls in both the modes are well within the normality bounds. In fact, the spatiotemporal signatures of the two cohorts almost overlap with each other.

### Decision Models

**Figure 6.**
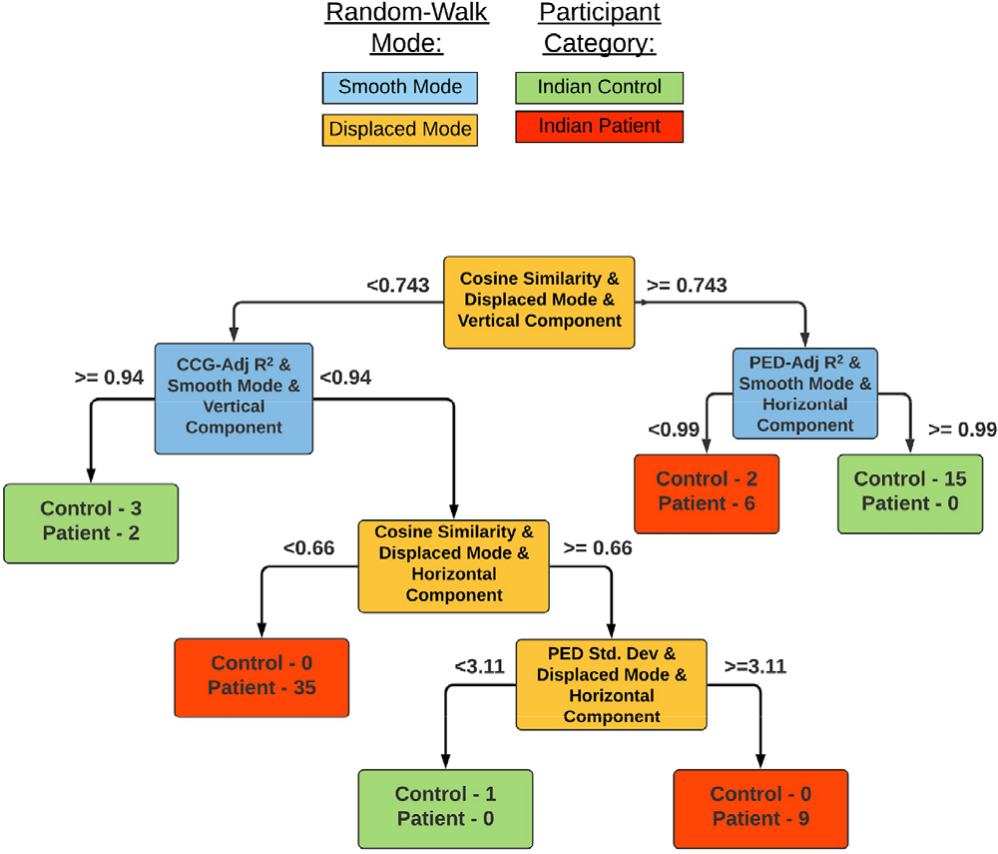
Decision tree model for screening patients from healthy controls. The terminal nodes at each level of the tree indicate the number of correctly classified and misclassified (if any) subjects.

Figure 6 shows the “Screening” Model, that is, the decision tree model that was constructed to separate the healthy from either of the ophthalmic disorders in our group based on their EM. To understand how the decision tree works, we provide some explanation. At this stage, it is assumed that a participant has performed both the “smooth” and “displaced” variant of the experiment. For easy readability, we have color coded “smooth” mode features in blue and “displaced” mode features in orange.

**Figure 7.**
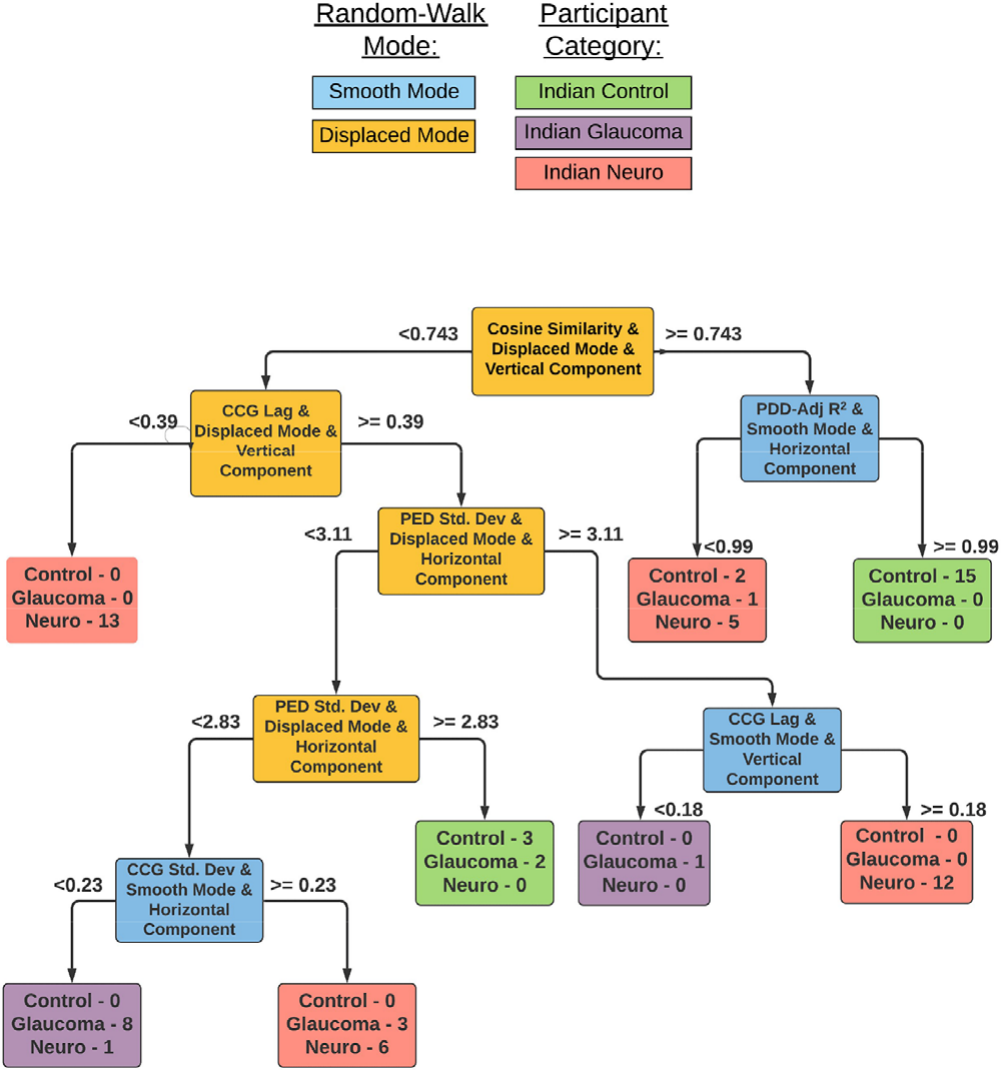
Decision tree model for screening the individual patient groups from healthy controls.

The model first checks for the *cosine similarity* of the vertical eye movements in the “displaced” mode of the experiment. This is called the “root” of the decision tree and is indicative of the most useful feature in the entire list. If the similarity is more than 0.74, then the model subsequently checks for the *variance explained* by the positional error distribution in the “smooth” mode. If this value is higher than 0.99, then the subject is classified as a “healthy” subject (coded in green). Similarly, other combinations can lead to “healthy” or “patient” (coded in red) categories—such terminal categories are called a “leaf.” Usually, a leaf is dominated by one of the classes; however, there can be a minority of the other class—these are misclassifications and affect the overall accuracy of the model. This model achieved a classifying accuracy of 94.5% after performing a “Leave-One-Out” cross-validation procedure. The model had a true-positive rate/sensitivity—a measure crucial in screening procedures—of 96% and a specificity of 90%. Figure 7 shows the decision tree with the useful features for individual categorization of a participant into one of three categories, i.e. healthy, glaucoma or neuro-ophthalmological disorder. This model achieved an accuracy of 86.3% (TPR for the neuro-ophthalmologic group = 97%, glaucoma = 60%, and controls = 86%).

### Evaluation of the Patient-Categorization Model

Table 5 shows the confusion matrix for the individual patient-categorization model shown in Figure 7. The values on the principal diagonal of the matrix represent the number of participants correctly classified in the specific category. Off-diagonal values represent the misclassifications. Table 6 shows the statistics of the confusion matrix such as the true- and false-positive results, and the true- and false-negative results as described in the preceding section. Table 7 shows additional performance metrics such as the sensitivity, specificity, PPV, and NPV for the model.

**Table 5.** The Confusion Matrix for the Individual Patient-Categorization Model

	Predicted Class		
True Class	Controls	Glaucoma	Neuro-Ophthalmology
Controls	18	—	3
Glaucoma	2	9	4
Neuro-Ophthalmology	—	1	36

**Table 6.** The Confusion Matrix Statistics Showing the Number of True- and False-Positives and True- and False-Negatives Across the Three Groups

Class	True Positives	False Positives	False Negatives	True Negatives
Controls	18	2	3	50
Glaucoma	9	1	6	57
Neuro-Ophthalmology	36	7	1	29

**Table 7.** Additional Performance Metrics Showing the Sensitivity (TPR), Specificity (TNR), Positive Predictive Value (Precision) and Negative Predictive Value For All the Three Groups

	Performance Metrics			
Class	Sensitivity	Specificity	Positive Predictive Value	Negative Predictive Value
Controls	86%	96%	90%	94.3%
Glaucoma	60%	98%	90%	90.4%
Neuro-Ophthalmology	97%	81%	83.7%	96.6%

## Discussion

The main findings of this study are that eye movement patterns of patients with glaucomatous and neuro-ophthalmologic VFD vary sufficiently from healthy people such that they can be used for screening purposes. The STP of our method describe their oculomotor behavior and agree well with the current literature on this. Moreover, these STP are stable across ethnicity. An explainable decision model based on these STP is able to classify the patients based on their EM behavior. Below, we describe these aspects in more detail.

### Screening Patients Based on the STP of Eye Movements

There is precedent in using EM to examine the effects of VFD – they have been applied in the context of driving,^20^ face recognition,^21^ and reading.^22^ Here, we attempt to screen patients based on their EM as well. The “screening” decision tree model (see Fig. 6) shows that the spatiotemporal feature *cosine similarity* in the “displaced mode” is the most critical feature in terms of discriminating the healthy from those who have either a glaucomatous or neuro-ophthalmologic VFD. Most of the patients (44/52) are categorized as such, based on a reduced cosine similarity in the “displaced mode.” In fact, the decision tree explains that both the horizontal and vertical eye positions in the “displaced mode” are dissimilar to that of the stimulus position as compared to those of controls. Furthermore, the decision tree selects two other distinguishing features: (1) the *adjusted* *R^2^* of the Gaussian fit model in the “smooth mode” average velocity cross-correlogram. (2) The *standard* *deviation* of the positional error distribution in the “displaced” mode. The former feature is an indication of how well the latency of smooth pursuit resembles a Gaussian whereas the latter describes the spatial uncertainty in the visually driven eye movements while searching for a blob that jumped randomly every two seconds. Overall, the decision model makes use of two properties from each of the “smooth” and the “displaced” modes to screen for patients.

### Oculomotor Behavior in Glaucoma

Our group of glaucoma patients had lower spatiotemporal fidelity as compared to that of controls, that is, with reduced *cosine similarity*. A closer look at the “Patient Categorization” decision tree model in Figure 7 reveals that glaucoma patients are categorized as such based on at least 3 “displaced mode” features, namely: *cosine similarity*, *temporal lag* of the average velocity cross-correlogram, and the *standard deviation* of the positional error distribution. This pattern is interpretable as glaucoma patients typically have peripheral visual field loss. Consequently, they would have had to make a large number of saccades to search for the luminance blob when it jumped to a new random location every two seconds. More specifically, the glaucoma group had both a higher *temporal lag* and *spatial uncertainty* when they had to make saccades as compared to the control and neuro-ophthalmologic disorder groups (these can be observed by tracing the decision paths leading to the glaucoma category - shown in violet). Such observations are reported in the literature as well—Kanjee et al.^23^ report that saccadic eye movements were significantly delayed in patients with early, moderate and advanced glaucoma. Najjar et al.^24^ also report that POAG patients had a much lower average saccade velocity as compared to controls when the visual target was presented in the peripheral region. Moreover, they observed that the saccades made by these patients were hypometric and had significantly reduced amplitude and gain as compared to controls. Another interesting observation is that the “smooth” mode is not a necessary condition for glaucoma categorization because the “displaced” mode features suffice to do so. Nevertheless, for clinical relevance, we note that during smooth pursuit, the glaucoma group had a worse time lag than the controls but did not differ much as compared to the neuro-ophthalmologic disorder group (see Fig. 4). Similar observations on smooth pursuit in glaucoma have been made wherein POAG patients watched a kinetic target and had impaired latency and accuracy of eye movements as compared to those of normal observers.^25^

### Oculomotor Behavior in Neuro-Ophthalmological Disorders

The neuro-ophthalmology disorder group also had lower spatiotemporal fidelity as compared to the controls. However, what sets it apart from the glaucoma group is the fact that they showed higher temporal lags than the glaucoma group during smooth pursuit. These observations can be expected as patients with unilateral cerebral lesions may have deficient smooth pursuit when stimuli targets move towards the site of lesion.^26^^,^^27^ Meanwhile, in the “displaced mode,” the glaucoma group of patients had higher lags as compared to those of the neuro-ophthalmologic group. About half of the participants under the neuro-ophthalmic group had higher standard deviations in the positional error distribution for the “displaced mode.” This indicates that spatial uncertainty is higher while performing saccades—which is consistent with the notion that saccadic dysmetria is likely to occur primarily for moving targets.^28^^,^^29^ The temporal lag and uncertainty during smooth pursuit are also higher than the rest for at least half of the participants in the group. This pattern can be attributed to the fact that temporal uncertainty increases as the complexity in the eye movement planning is under risk due to the increase in brain injury besides age. Moreover, an increase in mean latency also increases the standard deviation of the eye movement duration.^30^

### STPs of EM are Stable Across Indian and Dutch Controls

Studies have shown that eye tracking precision can vary for different eye colors^31^^,^^32^; for example, eye trackers typically respond with lower precision and accuracy for subjects with bluish eyes as compared to brownish ones.^33^ Consequently, we wanted to check whether a combination of ethnicity and eye tracking setup would affect the STP of EM. However, as seen in Figure 5, both the control cohorts are similar in terms of the STP of their EM. We can infer two things from the result: (1) The EM patterns of the two ethnic groups were similar in response to the stimuli shown. (2) Our method accounts for at least some differences in eye tracking setups—such as screen size, monitor refresh rate, and viewing distances. The latter suggests that our method may be used across different populations and in different eye tracking setups.

### Using Decision Trees as Clinically Explainable Machine Learning Models

There are three main reasons why, at present, DTs are our preferred choice of classification: (1) Although deep learning methods far exceed human performance when it comes to classification tasks, their training requires using enormous datasets. Moreover, they are considered as “black-box” models with no explicit knowledge representation. Although such approaches are generally fine for tasks such as natural image or text classification, it is crucial to have “explainable artificial intelligence (XAI)” models when it comes to the medical field.^34^ Because a clinician could be asked by a patient to explain why they took a medical decision, it would be eventually imperative for them to explain and retrace an AI-assisted clinical decision. Given that we have a limited amount of data and current deep learning-based XAI-approaches are still under development, we used a relatively uncomplicated and explainable technique of DTs. (2) DTs, by design, are able to perform feature selection and consequently reduce the number of features to be considered.^35^ (3) DTs have an added advantage of not requiring distributions or prior probabilities associated with different classes (in our case, associated with glaucoma, neuro-ophthalmologic disorder or control groups).

## Limitations and Future Directions

There are some limitations to our current study. We used a blob with fixed size and luminance as the target stimuli in our experiment. Now, the ratio of the background luminance to the target luminance—also known as “differential light sensitivity” (DLS)—is related to the receptive field size of the ganglion cells.^36^ For small stimuli, the DLS is linearly related to the stimulus size because the human visual system summates the luminance information. However, in our case, there are two issues: First, we used a Goldman size III blob, and for these larger sizes, the luminance information summation is incomplete and depends on the retinal eccentricity.^37^ Second, visual sensitivity decreases almost linearly with age.^38^ Consequently, it would be useful to investigate the effects of adaptive size (as a function of eccentricity), luminance, and extent of the blob jump in the “displaced mode” on the screening results of our approach.

Another limitation is that currently, our approach does not provide visual field maps such that they can be compared directly with those provided by the SAP. However, such maps would typically require a large corpus of normative data, which is beyond the scope of this study. Moreover, we stress the fact that at present, our approach is to be considered primarily as a screening tool. In other words, to diagnose patients still requires considering other sources of information as well, such as ocular pressure, fundus photography, or optical coherence tomography images.

Although we tested the stability of STP of EM on two ethnic groups (Indian and Dutch), future studies could consider testing larger sample sizes and covering more ethnic groups to verify the robustness of the screening results. Future studies could also consider comparing our method with other recent screening methods—for example, fundus photo-based artificial intelligence systems^39^^,^^40^ have shown promising results in the context of glaucoma screening. Tablet^41^ and virtual reality–based^42^ perimetric approaches have also been developed and should be compared to EM-based approaches.

## Conclusions

EM made in a continuous tracking paradigm can be used as a basis to screen for the underlying VFD. We find that the STP of EM are unique to patients with glaucomatous VFD, neuro-ophthalmological VFD and healthy controls, respectively. The method also corroborates well with the clinical characteristics of glaucoma and neuro-ophthalmologic disorders. Moreover, the EM parameters are stable across ethnicity and two eye tracking setups. Consequently, our method may be used across different ethnicities and eye tracking equipment. Our approach eliminates the need for manual responses, such as required as in SAP and FDT. We conclude that the test has clear potential as a screening tool in clinical practice, particularly in groups unable to perform SAP.
